# Effects of adolescent oxycodone exposure on adult remifentanil self-administration and gene expression in male and female mice

**DOI:** 10.3389/fnbeh.2026.1984313

**Published:** 2026-10-28

**Authors:** Anna K. Leonard, Kyle A. Windisch, Keegan S. Krick, Marisol Hooks, Shuo Zhang, Julie A. Blendy, Elizabeth A. Heller

**Affiliations:** 1 Department of Systems Pharmacology and Translational Therapeutics, University of Pennsylvania, Philadelphia, PA, United States; 2 Epigenetics Institute, Perelman School of Medicine, University of Pennsylvania, Philadelphia, PA, United States

**Keywords:** adolescent, intravenous self-administration (IVSA), mice, oxycodone, remifentanil, RNA-Seq

## Abstract

**Introduction:**

The Opioid Epidemic is a public health crisis in the United States, contributing to an estimated 54,000 deaths in 2024. A major contributing factor is prescription and recreational use of painkillers such as oxycodone. Adolescent painkiller use predicts later-life transition to more potent opioids, but the causal relationship and its neurobiological mechanisms are not fully understood.

**Methods:**

Adolescent male and female mice were exposed to oxycodone (OXY) or saline (SAL), then tested for intravenous self-administration (IVSA) of remifentanil, a potent synthetic opioid, in adulthood. RNA sequencing (RNA-seq) was performed on reward-related brain regions, the nucleus accumbens (NAc) and ventral tegmental area (VTA), from adult mice that underwent adolescent OXY or SAL with or without subsequent IVSA.

**Results:**

Adolescent OXY increased adult remifentanil IVSA in females but not males. In contrast to the behavior, adolescent OXY induced modest differential gene expression in adulthood, though several interesting candidate genes were identified for future study, particularly in the male NAc. We further found that adolescent OXY induced differential splicing in all comparisons, with a potentially greater response in the female VTA post-IVSA. Gene-Set Enrichment Analysis (GSEA) showed that adolescent OXY treatment (vs. SAL) produced gene-set enrichment in the adult male NAc, which was similar whether or not animals later underwent IVSA. In the female NAc, gene-set enrichment was minimal with OXY treatment alone, while the IVSA cohort showed substantial enrichment. In contrast, adolescent OXY exposure alone was associated with similar patterns of gene-set enrichment in the male and female VTA, which was absent in the IVSA cohort. Complementary Rank-Rank Hypergeometric Overlap (RRHO) analysis identified patterns of gene expression concordance that recapitulated the GSEA results.

**Discussion:**

Considering the IVSA and RNA-seq together, these data lead us to new hypotheses that male mice mount a gene expression response in the NAc following adolescent OXY associated with a lack of long-term increases in volitional drug taking. In contrast, females may exhibit an alternative splicing response in the VTA during adult IVSA which is associated with increased drug taking. These analyses have provided valuable targets for future validation and mechanistic investigation of these new hypotheses.

## 1 Introduction

The opioid epidemic is a serious public health crisis in the United States. From 1999 to 2004, per capita rates of opioid overdose increased approximately sixfold, contributing to an estimated 54,000 deaths in 2024 (Garnett and Minino, 2024; Hedegaard et al., 2021; Spencer et al., 2022). While potent illicit synthetic opioids like fentanyl are currently the major drivers of opioid-related overdose deaths, painkillers such as oxycodone remain the most commonly misused opioids (Centers for Disease Control and Prevention, 2023). Adolescents are a population of particular concern regarding painkiller use and misuse. The use of drugs such as oxycodone is frequently initiated in adolescence following life events such as surgeries or sports injuries (Sutherland et al., 2026; Levy et al., 2026). While the overall rate of meeting opioid-use disorder (OUD) criteria is low in adolescents (1–2%), the Youth Risk Behavioral Survey reports that 12% of adolescents have ever used an opioid not as prescribed (Centers for Disease Control and Prevention). Importantly, both prescribed use and misuse of painkillers in adolescence predict later-life misuse and transition to more potent drugs (Cerdá et al., 2015; Miech et al., 2015), but the causal relationship is not fully understood.

A driving hypothesis of our research is that neurobiological correlates of prolonged drug abstinence reflect a homeostatic process that works to restore baseline drug-related behavior. A substantial body of literature, including our prior work, supports the idea that drugs of abuse induce changes in neuronal gene expression in reward-related brain regions, many of which emerge only during prolonged abstinence and involve genes distinct from those regulated by intoxication and acute withdrawal (Feng et al., 2014; Feng et al., 2024; Carpenter et al., 2020; Carpenter et al., 2021; Sanchez et al., 2016; Xu et al., 2021; Sadakierska-Chudy et al., 2017; Freeman et al., 2008; Martínez-Rivera et al., 2025). These regions include the Ventral Tegmental Area (VTA), which is disinhibited by exogenous opioids to increase downstream dopamine release into the mesolimbic reward system, and the nucleus accumbens (NAc), a major downstream target of these dopaminergic VTA projections and a key regulator of reward-directed behavior (Koob and Volkow, 2010). Although this literature is extensive for cocaine, studies of opioid-induced gene expression are more limited (Browne et al., 2025; Browne et al., 2023; Magnard et al., 2026). Several studies have reported differential behavioral and transcriptomic responses to opioid exposure in adolescent vs. adult rodents (Zhang et al., 2009; Koek et al., 2012; Niikura et al., 2013; Koek, 2014; Mayer-Blackwell et al., 2014; Zhang et al., 2015), but none have used next-generation sequencing (NGS) to characterize the genome-wide transcriptomic response to adolescent opioid exposure.

To examine the long-term effects of adolescent opioid exposure, we developed a model in which adolescent mice receive oxycodone via subcutaneous osmotic minipumps for 28 days (d) from postnatal day (PND) 28–56, followed by a 28-d drug-free period (PND 56–84). This is referred to throughout this article as “OXY,” and saline minipump controls are referred to as “SAL.” We previously reported that morphine conditioned place preference (CPP) is increased when mice undergo this OXY paradigm in adolescence, but not in adulthood (Sanchez et al., 2016). We also found, using targeted gene expression profiling, that adult OXY increased the expression of dopamine signaling-related genes in the VTA at this timepoint, while adolescent OXY did not (Carpenter et al., 2021). Together, these data lead us to the hypothesis that abstinence-induced homeostatic gene expression is disrupted in adolescent mice, contributing to the long-term behavioral effects of OXY exposure. A major goal of the current study was to characterize the effect of adolescent OXY on intravenous self-administration (IVSA) of the fentanyl analog, remifentanil. Whereas CPP models drug reward, IVSA models volitional drug-taking and drug reinforcement (Green and Bardo, 2020). This design serves as a highly face-valid, translationally relevant model of painkiller exposure in adolescence followed by a transition to voluntary use of more potent opioids later in life. We hypothesized that adolescent OXY would increase adult remifentanil IVSA in both males and females. A second major goal was to characterize genome-wide gene expression changes in the NAc and VTA following adolescent OXY using RNA sequencing (RNA-seq). We also sequenced tissue collected from mice that underwent remifentanil IVSA. As it has been reported that prior drug exposure can alter the brain’s transcriptional response to re-exposure (Emerson et al., 2023; Browne et al., 2023), we hypothesized that adolescent OXY would modulate the response to remifentanil IVSA.

In addition to analyzing differential gene expression, we analyzed differential alternative splicing (AS) between OXY and SAL mice. It has been well established that cocaine produces robust differential AS in genes distinct from those showing differential expression (Xu et al., 2021; Feng et al., 2014), but little research has focused on AS in opioids except with regard to putative alternative splicing of the opioid receptor *(Opr)* genes (Donadoni et al., 2022; Brown et al., 2022; Liu et al., 2021; Bhatnagar and Heller, 2025).

Next-generation sequencing is a powerful tool for target discovery, which we have previously leveraged to identify genes exhibiting differential expression or alternative splicing in response to drug exposure and abstinence (Winter et al., 2025; Xu et al., 2021). We have further demonstrated through exogenous manipulation that these genes are causally relevant to drug reward and drug seeking. That said, focusing on differential expression or splicing of single genes may miss changes in broader biological pathways, as single genes rarely act in isolation to influence cell function. Thus, in addition to searching for individual DEGs between OXY and SAL groups, we leveraged multiple threshold-free bioinformatic tools—gene-set enrichment analysis (GSEA) (Subramanian et al., 2005; Xu et al., 2024; Wu et al., 2021) and rank-rank hypergeometric overlap (RRHO) (Cahill et al., 2018; Plaisier et al., 2010)— to identify patterns in gene expression and biological term enrichment across sexes, timepoints, and brain regions. Through this work, we uncovered striking sex-specific effects of adolescent OXY on adult remifentanil IVSA, and potential sex- and brain-region-specific transcriptional responses in the NAc and VTA. These data have generated new hypotheses about the sex-specific response to adolescent opioid exposure and provided candidate genes for study in our future work.

## 2 Methods

### 2.1 Animals

C57Bl/6NTac mice (B6; Taconic, Hudson, New York) were bred in-house at the University of Pennsylvania. Male and female offspring were weaned at postnatal day (PND) 21. Same-sex littermates were group-housed (2–4 per cage) with ad libitum access to food and water under a 12-h light cycle for the duration of the experiment. All procedures were approved by the University of Pennsylvania Animal Care and Use Committee and were in compliance with the National Institutes of Health Guide for the Care and Use of Laboratory Animals.

#### 2.1.1 Anesthesia and euthanasia

For all surgical procedures described below, anesthesia was induced with 4% isoflurane in an induction chamber and maintained at 1–3% via nosecone. Perioperative meloxicam (5 mg/kg s.c.) was administered for analgesia. For tissue dissection, euthanasia was prepared by rapid live cervical dislocation conducted by trained personnel.

### 2.2 Adolescent oxycodone exposure

The timeline of oxycodone exposure via subcutaneous minipump is outlined in Figure 1A. A flowchart of all mice enrolled in the study is provided in Supplementary Figure 1A. All surgical procedures were conducted using aseptic technique. At PND 28, all mice were implanted with subcutaneous osmotic minipumps (model 1,004; Alzet, Cupertino, CA) placed parallel to the thoracic vertebrae. Minipumps delivered oxycodone HCl (Spectrum Chemical, New Brunswick, NJ) dissolved in sterile 0.9% saline at an average dosage of 3 mg/kg/day for 28 days (PND 28–56), or saline only for control mice. This timeline spans nearly the complete period of adolescent development in mice, which consists of “early” (PND21-33), “mid” (PND 34–46), and “late” (PND 47–59) phases (Adriani et al., 2002; Hodgson et al., 2010; Hodgson et al., 2009). Dosage was based on the typical average weight of our B6 litters at the midpoint of administration (6 weeks of age; M = 20 g, *F* = 17 g) (Sanchez et al., 2016). Mice were randomly assigned to OXY or SAL groups at weaning, with same-sex littermates split across treatment groups. After 28d of exposure, minipumps were surgically removed. Mice then underwent a 28-d drug-free period (PND56-84) in the home cage to reach adulthood. At this point, mice underwent intravenous self-administration (IVSA) testing or were dissected for RNA-seq. Throughout this article, the complete timeline of 28 d oxycodone exposure + 28 d drug-free is referred to as “OXY,” with saline controls referred to as “SAL.”

**Figure 1:**
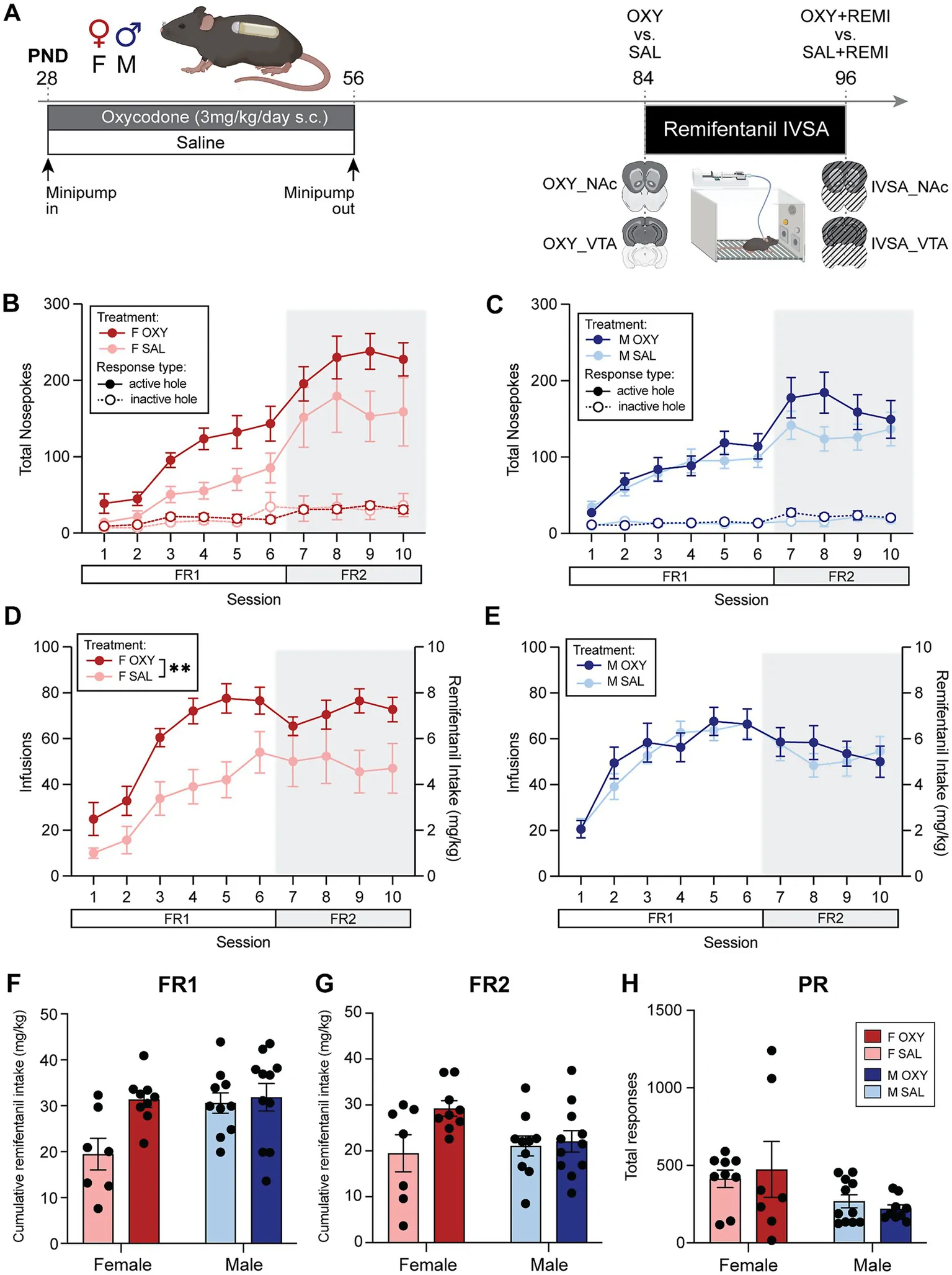
Adolescent OXY elevated adult fixed-ratio remifentanil intake in female but not male mice. **(A)** Male (M) and female (F) C57BL/6 mice were exposed to oxycodone (average 3 mg/kg/day) or saline via subcutaneous minipump from PND 28–56, followed by a 28-d drug-free period from PND 56–84. At PND 84, a subset of mice was sacrificed for dissection of the NAc and VTA for RNA-seq (OXY vs. SAL). Additional mice proceeded through remifentanil IVSA and were sacrificed immediately following the final session of IVSA (OXY + REMI vs. SAL + REMI). **(B)** Mean daily active and inactive nose pokes in F OXY mice (*n* = 9) compared to F SAL (*n* = 7). **(C)** Mean daily active and inactive nose pokes in M OXY (*n* = 11) compared to M SAL (*n* = 10). **(D)** Mean daily remifentanil infusions (left *Y* axis) and extrapolated intake (right *Y* axis) in F OXY mice compared to F SAL. **(E)** Mean daily remifentanil infusions (left *Y* axis) and extrapolated intake (right *Y* axis) in M OXY compared to M SAL mice. **(F)** Mean cumulative remifentanil intake during fixed ratio 1 (FR1). **(G)** Mean cumulative remifentanil intake during FR2. **(H)** Mean total nose pokes during progressive ratio (PR). All data are shown as group mean +/− SEM. **(F,G)** Data points represent individual mice. *p < 0.05, **p < 0.01. Complete ANOVA and *t*-test statistics are provided in Supplementary Table 1. Schematics in A adapted from BioRender. Leonard, **A**. (2026) https://BioRender.com/s6f3ij3.

### 2.3 Intravenous self-administration (IVSA)

#### 2.3.1 Jugular catheter surgeries

A subset of mice that underwent OXY or SAL treatment (initial *n* = 12 mice/treatment/sex; Supplementary Figure 1A) began remifentanil IVSA 28d following minipump removal (Figure 1A; OXY + REMI or SAL + REMI). One week prior to catheter surgeries, mice were moved to the IVSA suite, a dedicated satellite housing room on a reverse light–dark cycle, to habituate. Five days prior to the first IVSA session, mice were implanted with indwelling jugular catheters. Skin was shaved over the dorsal mid-scapular region and at the neck over the right jugular vein and cleaned with alternating ethanol and betadine. A 1 cm incision was made in each location, and a sterile silastic catheter was fed subcutaneously from the mid-scapular incision to the neck. The jugular vein was exposed and punctured with a sterile needle, and the catheter was inserted into the vein and sutured in place. Both incisions were closed with non-absorbable sutures. Catheters were flushed daily with gentamicin dissolved in heparinized saline and were sealed with a dummy cap when not in use. Following a 5-day post-operative recovery, IVSA testing was carried out during the dark cycle.

#### 2.3.2 IVSA testing

Mice were placed in operant chambers (Med Associates) for daily 2-h intravenous (i.v.) remifentanil (0.1 mg/kg/infusion) self-administration sessions under a fixed ratio (FR) 1 schedule, with one active hole nose poke triggering one infusion. After each drug infusion, there was a 20-s timeout period during which the responses had no consequences. Mice were limited to a maximum of 100 remifentanil infusions per daily session. Remifentanil is a potent synthetic opioid that is rapidly metabolized and has a short half-life (3–10 min) (Sreevastava and Bhargava, 2004), which promotes operant learning and allows mice to titrate their intake. The intravenous dose of remifentanil used (0.1 mg/kg/infusion) maintains the highest reinforcer intake on a dose–response curve in mice (Slosky et al., 2022). After six sessions of FR1, the response requirement was increased to FR2 for four sessions. Mice then had a single 4-h progressive ratio (PR) session to assess motivation for remifentanil using 𝑈(𝑛) = 5𝑒0.2𝑛 −5 to calculate the nose poke requirement for each subsequent infusion. At the end of the PR test, catheter patency was tested via administration of 0.05 𝜇l ketamine (2 mg/mL). Only patent mice, defined as a loss of muscle tone within a few seconds after i.v. ketamine, were included in subsequent behavioral and RNA-seq analysis (Supplementary Figure 1A). Patent mice completed one additional FR2 session the following day, followed by sacrifice and dissection immediately afterward (PND 96).

#### 2.3.3 Statistical analysis of behavior

Statistics were analyzed and plotted in GraphPad Prism Version 11.0.2. All behavioral data are shown as mean +/− standard error of the mean (SEM). Daily total active and inactive nose pokes and daily total infusions earned were compared by three-way (sex × treatment × session) repeated-measures ANOVA with Geisser–Greenhouse correction, where treatment = OXY vs. SAL. When sex x treatment interaction terms were significant (*p* < 0.05), post-hoc two-way (treatment x session) ANOVAs were performed within sex. Daily remifentanil intake was computed as daily earned infusions * dose (0.1 mg/kg/infusion). Cumulative intake was calculated as the sum of daily intake across FR1 (sessions 1–6) and FR2 (sessions 7–10). The progressive ratio was assessed by the total number of active nose pokes, total number of infusions earned, and breakpoint (number of responses required for the final earned infusion). Cumulative intake and progressive ratio metrics were compared by two-way (sex × treatment) ANOVA.

### 2.4 Tissue collection

Brain tissue for RNA-seq was collected from a subset of mice following the 28-d drug-free period after minipump removal (OXY vs. SAL; *n* = 8 mice/treatment/sex). This timepoint is referred to throughout the sequencing results as ‘OXY’ (Table 1 and Supplementary Figure 1). Mice that proceeded through IVSA were sacrificed immediately following the final session as described above (Figure 1A; OXY + REMI vs. SAL+REMI); this timepoint is referred to throughout the sequencing results as ‘IVSA’ (Table 1). Mice were euthanized by cervical dislocation, and brains were rapidly removed and washed in ice-cold phosphate-buffered saline with a protease inhibitor cocktail (Roche cOmplete). One-millimeter sections of the nucleus accumbens (NAc) and ventral tegmental area (VTA) were prepared with a coronal tissue matrix, and regions were bilaterally microdissected with a 2 mm (NAc, Figure 2A) or 1.5 mm (VTA, Figure 2C) diameter punch. Tissue was immediately frozen on dry ice and stored at −80° C until downstream analysis.

**Table 1:** Nomenclature for RNA-seq datasets.

RNA-SEQ Batch:	n	Sex	Timepoint	Brain Region
F_OXY_NAc	4 OXY, 4 SAL	Female	‘OXY’: OXY vs. SAL	Nucleus accumbens
F_IVSA_NAc	3 OXY, 4 SAL		‘IVSA’: OXY + REMI vs. SAL+REMI	
M_OXY_NAc	4 OXY, 4 SAL	Male	‘OXY’: OXY vs. SAL	
M_IVSA_NAc	3 OXY, 4 SAL		‘IVSA’: OXY + REMI vs. SAL+REMI	
F_OXY_VTA	4 OXY, 4 SAL	Female	‘OXY’: OXY vs. SAL	Ventral tegmental area
F_IVSA_VTA	3 OXY, 3 SAL		‘IVSA’: OXY + REMI vs. SAL+REMI	
M_OXY_VTA	3 OXY, 3 SAL	Male	‘OXY’: OXY vs. SAL	
M_IVSA_VTA	4 OXY, 4 SAL		‘IVSA’: OXY + REMI vs. SAL+REMI	

**Figure 2:**
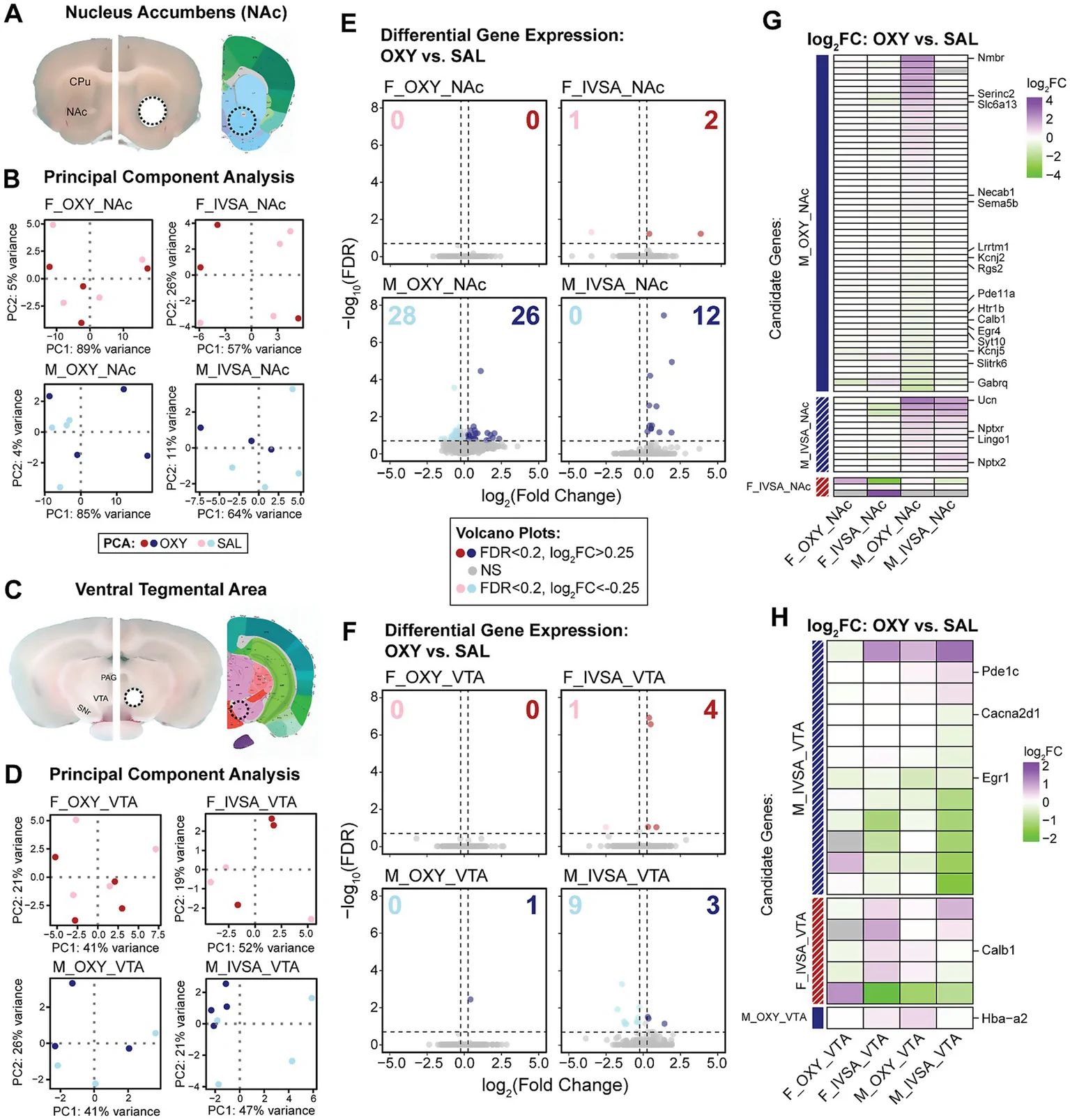
Adolescent OXY induced modest differential gene expression in the male and female NAc and VTA. **(A)** Representative image of a NAc tissue section and the same section (mirrored) with a 2 mm tissue punch (dotted line), compared to the Allen Brain Atlas annotation of NAc (Allen Institute for Brain Science, 2004, https://atlas.brain-map.org). **(B)** Principal Component Analysis (PCA) of VST-transformed length-scaled gene expression of the top 500 most variable genes for all RNA-seq datasets in the NAc. F_OXY_NAc: *n* = 4 OXY, 4 SAL; M_OXY_NAc: *n* = 4 OXY, 4 SAL; F_IVSA_NAc: *n* = 3 OXY, 4 SAL; M_IVSA_NAc: *n* = 3 OXY, 4 SAL. **(C)** Representative image of a VTA tissue section and the same section (mirrored) with a 1.5 mm tissue punch of VTA, compared to the Allen Brain Atlas annotation of VTA. **(D)** PCA of VST-transformed length-scaled gene expression of the top 500 most variable genes for all RNA-seq datasets in the VTA. F_OXY_VTA: n = 4 OXY, 4 SAL; M_OXY_VTA: n = 3 OXY, 3 SAL; F_IVSA_VTA: n = 3 OXY, 3 SAL; M_IVSA_VTA: n = 4 OXY, 4 SAL. **(E,F)** Volcano plots of log_2_FC vs. -log_10_FDR in OXY vs. SAL mice in NAc **(E)** and VTA **(F)** datasets, computed by DESeq2. Highlighted genes meet cutoffs of FDR < 0.2 and log2FC > |0.25|. Labels indicate the total number of positive and negative genes meeting these cutoffs for each dataset. **(G,H)** For all candidate genes (FDR < 0.2, log2FC > |0.25|) identified in any NAc **(G)** or VTA **(H)** dataset, log_2_FC is shown across all datasets within each brain region. Labels and bars on the far left indicate which dataset each set of genes originates from (solid blue = M_OXY, solid red = F_OXY, striped blue = M_IVSA, striped red = F_IVSA). Select genes with known neuronal function are labeled on the right. Log_2_FC and FDR for labeled genes are provided in Table 2.

### 2.5 RNA extraction

Due to their small size, bilateral 1.5 mm VTA punches were pooled (n = 2 mice per sample) to increase RNA yield. Bilateral 2 mm NAc punches were processed individually. A flowchart of samples analyzed or excluded from analysis is shown in Supplementary Figure 1B. Samples were batched by sex, timepoint, and brain region for RNA extraction and sequencing, with a final *n* = 3–4 samples per treatment (OXY vs. SAL) per batch (Table 1). This design maximizes our ability to directly statistically compare OXY vs. SAL groups in the resulting datasets despite physical limitations on the number of samples that could be processed at one time. Sexes, brain regions, and timepoints were compared only through visualization of patterns across datasets. *N* = 3–4 per group is consistent with other exploratory and hypothesis-generating RNA-seq studies in prior publications in the field (Walker et al., 2018; Bagot et al., 2016; Kwapis et al., 2018). Within our lab, this is consistent with the n typically used for exploratory RNA-seq studies (Murphy et al., 2026; Hokenson et al., 2026), including those that generated targets that were later functionally validated *in vivo* (Xu et al., 2021; Carpenter et al., 2020).

Bulk RNA-seq was performed on RNA extracted from the cytosolic fraction of tissue samples. Tissue was first separated into nuclear and cytosolic fractions in 500ul Nuclear Extraction Buffer (20 mM HEPES-KOH, 10 mM KCl, 20% glycerol, 0.1% Triton-X 100, 0.5 mM spermidine, 1X Roche cOmplete Protease Inhibitor Cocktail) in a Dounce homogenizer. Nuclei were pelleted by spinning at 600 rcf for 10 min at 4° C. The supernatant containing cytosolic RNA was immediately mixed with 1 mL Trizol and frozen at −80° C overnight. Samples were then thawed, mixed with 200 μl chloroform, and incubated at RT for 3 min with shaking. The liquid phase containing RNA was separated by spinning at 12,000 rcf for 15 min at 4° C, then reserved in a new tube and mixed with 20 μg glycogen. RNA was precipitated by mixing with 500 μl isopropanol and pelleted by spinning at maximum speed for 10 min at 4° C. Pellets were washed twice in 1 mL 75% ethanol, dried, and resuspended in 30ul nuclease-free water.

### 2.6 Sequencing

Isolated RNA was sent to Azenta Life Sciences for paired-end Illumina sequencing. RNA was first assessed on an Agilent TapeStation. Samples were then library prepped with Poly(A) selection and sequenced on an Illumina NovaSeq (2 × 150 bp) at an estimated 50 million (50 M) reads per sample, a depth sufficient for both differential expression and alternative splicing analysis. Prior to analysis, RNA-seq libraries were evaluated using sequencing quality metrics from FastQC and summarized by MultiQC (Supplementary Table 1). All samples included in the final analysis passed internal quality control metrics at Azenta Life Sciences and met our thresholds for library size (≥40 million paired-end reads) and mean per-base quality score (≥Q35). Adapter content was trimmed using the bbduk function (BBTools package, link) with the included adapters.fa file to remove 3′ sequencing adapters and low-quality bases (Q < 30; ktrim = r k = 23 mink = 11 hdist = 1 qtrim = r trimq = 30) from the 3′ end.

#### 2.6.1 Differential gene expression analysis

Sequencing results were divided into eight datasets based on the sample processing batches, with each batch including a single sex (F or M), timepoint (OXY or IVSA), and brain region (NAc or VTA), and containing *n* = 3–4 samples per treatment group (OXY vs. SAL; Table 1 and Supplementary Figure 1B). Datasets are hereafter designated as Sex_Timepoint_Region, as outlined in Table 1.

Trimmed FASTQ files were pseudoaligned to a transcriptome index generated from the RefSeq annotation (GCF_000001635.27) (O’Leary et al., 2016) using kallisto (Bray et al., 2016). All samples met our predefined threshold for the proportion of reads pseudoaligned to the transcriptome (>85%). Output abundance.tsv files were imported into R using tximport (Soneson et al., 2015), and gene-level length-scaled count estimates were generated using $\text{countsFromAbundance}{=}^{“}\text{lengthScaledTP}M^{"}$ . Subsequent analysis was performed with DESeq2 (Love et al., 2014). Genes were retained for analysis if they had ≥10 length-scaled counts in at least three samples within their respective sequencing dataset.

Prior to differential expression analysis, variance stabilizing transformation (VST)-transformed gene expression was visualized by principal component analysis (PCA), hierarchical clustering, and sample-to-sample correlation using the top 500 most variable genes. Libraries exhibiting aberrant expression profiles across multiple measures, including separation from the remaining samples in PCA, formation of distinct clusters in hierarchical clustering, and reduced expression correlation with the remaining samples, were excluded from downstream differential expression and alternative splicing analysis (*n* = 1 library each in F_IVSA_NAc, F_IVSA_VTA; Supplementary Figures 1B–H). The final biological n for all analyses is outlined in Table 1.

Differential gene expression was quantified between OXY and SAL treatment within each dataset using a design formula of ~treatment . Statistical thresholds for visualization were defined as absolute log2 fold change (FC) > 0.25 and FDR < 0.2. Due to the higher odds of false-positive discoveries at this threshold, we define these as “candidate genes” of interest for validation and future study rather than presenting genes as definitively changing with treatment. The signed Wald statistic generated by DESeq2 was used to rank genes for threshold-free gene-set enrichment analysis, performed with clusterProfiler::gseGO (Wu et al., 2021), and stratified rank-rank hypergeometric overlap, performed with RRHO2 (Cahill et al., 2018) in R. Plots were generated in R with ggplot2 and pheatmap, with final figure modifications performed in Adobe Illustrator.

#### 2.6.2 Differential alternative splicing analysis

Trimmed FASTQ files were aligned to the mm39 genome using the splice-aware aligner STAR (Dobin et al., 2013) and the GENCODE comprehensive gene annotation vM36 (Mudge et al., 2025). Mapped reads were processed using RNA Multivariate Analysis of Transcript Splicing (rMATS) v4.3.0 to quantify alternative splicing events genome-wide (Shen et al., 2014). JCEC output files, which include both junction reads and on-target exonic reads to define PSI values, were used for all downstream visualization and analysis. Expressed splice junctions were defined as those with at least ten junction reads (sum of included and excluded junction counts) in at least three samples within each dataset. Differentially spliced genes (DSGs) were defined as genes with at least one significant event across all types with delta Percent Spliced In (dPSI) > |0.1| and FDR < 0.05. Volcano plots were generated in R with ggplot2. The overlap of DSGs with candidate genes from differential expression analysis was calculated with eulerr (Larsson and Gustafsson, 2018). Gene Ontology (GO) Overrepresentation Analysis (ORA) was performed with clusterProfiler::enrichGO , with the background gene set defined as genes with at least 1 TPM in at least three samples in each dataset. GSEA was performed with clusterProfiler::gseGO , with events ranked by dPSI∗log10(FDR).

## 3 Results

### 3.1 Adolescent OXY elevated adult fixed-ratio remifentanil intake in female but not male mice

The timeline of adolescent oxycodone exposure is outlined in Figure 1A. Male and female adolescent mice were treated with oxycodone or saline for 28 days from PND 28–56, followed by 28 days drug-free from PND 56–84 to reach adulthood. Both oxycodone-treated (OXY) and saline control (SAL) mice were then trained to perform remifentanil self-administration (IVSA).

Complete IVSA statistics are provided in Supplementary Table 2. All groups showed the expected discrimination between active and inactive nose pokes (Figures 1B,C). Additionally, a three-way sex × treatment × session ANOVA on daily active nose pokes revealed a main effect of treatment (*p* = 0.013). Although the sex x treatment interaction term was not significant for daily active nose pokes, mean daily active nose pokes were visually higher in female OXY vs. SAL mice across all sessions (Figure 1B), while male OXY mice had higher mean responses only during FR2 (Figure 1C). Furthermore, a three-way sex × treatment × session ANOVA on daily remifentanil infusions (Figures 1D,E) revealed a significant main effect of treatment (*p* = 0.0118), as well as significant interactions of sex × treatment (*p* = 0.034) and sex × session (*p* = 0.028). To further assess the interaction of sex × treatment on daily remifentanil infusions, *post-hoc* two-way treatment x session ANOVAs were performed within each sex. In females, there was a significant main effect of treatment (*p* = 0.006) on daily infusions earned (Figure 1D), while in males there was not (*p* = 0.731; Figure 1E). Together, these data demonstrate that adolescent OXY treatment elevates adult remifentanil self-administration on an FR schedule in female mice, but not in male mice.

Cumulative intake during FR1 and FR2 phases was assessed by two-way sex x treatment ANOVA. In FR1, there was a significant main effect of sex (*p* = 0.038) and treatment (*p* = 0.020), and a trending interaction (*p* = 0.057). As the interaction term narrowly missed the significance threshold, we conducted post-hoc testing using Šídák’s multiple comparisons test, which indicated a significant effect of treatment on cumulative FR1 intake in females (*p* = 0.012), but not in males (*p* = 0.923; Figure 1F). In FR2, there was a significant main effect of treatment (*p* = 0.042) and a trending interaction of sex x treatment (*p* = 0.094; Figure 1G).

In contrast to FR testing, progressive ratio (PR) testing showed a main effect of sex on total responses (*p* = 0.019; Figure 1H) and breakpoint (*p* = 0.019), with females having higher mean responses and breakpoints than males, but no significant main effect of treatment. PR infusions earned were not statistically different by sex or treatment. Together with the FR data, this suggests that while female adolescent OXY treatment results in increased remifentanil intake at a low fixed response requirement, it does not increase motivation to respond at this PR schedule.

### 3.2 Adolescent OXY induced modest differential gene expression in the adult NAc and VTA

Following from our behavioral results, we hypothesized that increased remifentanil IVSA in female mice after adolescent OXY would be associated with transcriptomic changes in the NAc and VTA. Further, as it has been reported that prior drug exposure changes the brain’s response to drug re-exposure (Emerson et al., 2023; Browne et al., 2025), we hypothesized that adolescent OXY would modulate the transcriptomic response to subsequent IVSA. Accordingly, we performed differential gene expression analysis (DESeq2) in the NAc and VTA between mice that received adolescent OXY vs. SAL, either with or without undergoing IVSA (Figure 1A and Table 1).

Samples were batched by sex, timepoint, and brain region for RNA-seq to prioritize the comparison of OXY vs. SAL, despite physical limits on the number of tissue samples that could be processed at one time. Corresponding datasets are referred to throughout the article as [sex-timepoint-brain region] (Table 1). In the dataset nomenclature, ‘OXY’ refers to the timepoint at the end of the 28d minipump exposure + 28d drug-free timeline (OXY vs. SAL; Figure 1A), which coincides with the first day of behavior in the IVSA cohort. ‘IVSA’ refers to the timepoint immediately following the final IVSA session (OXY + REMI vs. SAL+REMI, Figure 1A). Sex includes male (M) or female (F); brain regions include NAc (Figure 2A) or VTA (Figure 2C). Each sequencing batch contained *n* = 3–4 samples per treatment (OXY vs. SAL; Table 1 and Supplementary Figure 1B).

Normalized, VST-transformed gene expression was visualized by Principal Component Analysis (PCA) of the top 500 most variable genes. Across all datasets, samples did not cluster by treatment in either the NAc (Figure 2B) or VTA (Figure 2D), suggesting substantial between-animal biological variability. In line with the PCA results, differential gene expression computed by DESeq2 was modest across all datasets, with few genes surviving Benjamini-Hochberg correction for multiple comparisons (FDR). Dispersion estimates and MA plots for all DESeq2 analyses are provided in Supplementary Figure 2. The number of genes passing several thresholds for nominal p and FDR values is provided in Supplementary Table 3, along with DESeq2 output statistics for all genes passing nominal *p* < 0.05 in each dataset. FDR < 0.2 and |log2FC| > 0.25 were chosen as cutoffs for visualization in Figure 2, with the goal of identifying “candidate genes” for future validation, rather than presenting these individual genes as definitively changing with OXY treatment. Though less stringent than the typical cutoff of FDR < 0.05, this is more stringent than many studies in the field of substance-use disorder research, which often define differentially expressed genes (DEGs) as nominal *p* < 0.05 (Browne et al., 2023; Walker et al., 2018).

The limited differential gene expression in females was contrary to our hypothesis given the behavioral effect of adolescent OXY in females. The male NAc had the largest number of candidate genes (FDR < 0.2) identified (54 in M_OXY_NAc, 12 in M_IVSA_NAc; Figure 2E). We identified 0 candidate genes in F_OXY_NAc and 3 in F_IVSA_NAc. In the VTA, we identified 0 candidate genes in F_OXY_VTA, 5 in F_IVSA_VTA, 1 in M_OXY_VTA, and 12 in M_OXY_VTA (Figure 2F). While the confound of sex, timepoint, and sequencing batch limits our conclusions, these data lead us to a new hypothesis that the transcriptional response of the adult male NAc to adolescent OXY is associated with a lack of increased remifentanil intake vs. SAL controls.

There was no overlap of identified candidate genes between datasets in the NAc (Figure 2G) or VTA (Figure 2H). However, we were curious whether genes identified in each dataset might also exhibit expression changes in other datasets, despite not meeting the FDR cutoff. When we compared log_2_FC values for these genes across all datasets, patterns were visually apparent (Figures 2G,H). In general, candidate genes in M_OXY_NAc showed minimal log_2_FC in any other dataset. In contrast, genes with positive log_2_FC in M_IVSA_NAc also trended upward in M_OXY_NAc, despite not reaching our statistical cutoffs, suggesting that the transcriptomic response to adolescent OXY in the male NAc could be similar with and without IVSA exposure. Conversely, these genes trended downward in F_IVSA_NAc. A similar pattern was observed in the VTA: candidate genes in M_IVSA_VTA tended to change in the same direction, but with smaller magnitude, in M_OXY_VTA, whereas DEGs identified in F_IVSA_VTA tended to show the opposite direction of change in F_OXY_VTA. While our conclusions from these patterns are also limited by batch effects, these data lead us to an additional new hypothesis of a divergent transcriptional response to IVSA in the NAc between males and females with prior adolescent OXY.

Many candidate genes identified in M_OXY_NAc were neuronal, including potassium channels (*Kcnj2, Kcnj5*), calcium-binding proteins (*Necab1, Calb1*), synaptic proteins (*Syt10, Lrrtm1, C1ql2, Sema5b, Slitrk6, Sytl4, Serinc2*), neurotransmitter receptors (*Htr1b, Gabrq, Nmbr*), regulators of receptor-mediated signaling (*Rgs2, Pde11a*), a GABA transporter family member (*Slc6a13*), and an immediate early gene (*Egr4*). Those in M_IVSA_NAc were also neuron-related, including *Neuronal Pentraxin 2 (Nptx2)*, a synaptic protein that regulates excitatory synapse formation, and its receptor *Nptxr*; *Urocortin 1* (*Ucn*), a corticotropin-releasing hormone-related peptide; and *leucine-rich repeat and immunoglobulin-like domain-containing protein 1 (Lingo1)*, a negative regulator of neuron myelination that has previously been implicated as a driver of both neurodegenerative and neuropsychiatric disorders (Andrews and Fernandez-Enright, 2015). Candidate genes in M_IVSA_VTA also had clear neuronal relevance, such as *Early growth response protein 1* (*Egr1*), a classic immediate early gene; *Pde1c,* a calcium/calmodulin-stimulated cyclic nucleotide phosphodiesterase; and *Cacna2d1*, an auxiliary subunit of voltage-gated calcium channels. In females, one notable candidate gene in F_IVSA_VTA was Calbindin 1 (*Calb1;* log_2_FC = 0.334, FDR = 0.091), a calcium-buffering protein known to modulate dopamine signaling between the NAc and VTA (Brimblecombe et al., 2019). *Calb1* was also identified in M_OXY_NAc. The log_2_FC and FDR for the genes listed here are provided in Table 2. While these genes require further validation to clarify any causal role they may have in regulating volitional drug-taking after adolescent OXY, they all make compelling targets for our future work.

**Table 2:** Candidate genes of interest from differential expression analysis.

RNA-seq Dataset	Gene Symbol	log_2_FC	FDR
M_OXY_NAc	Slc6a13	1.118	3.61E-05***
	Necab1	0.310	0.010**
	C1ql2	−0.756	0.010**
	Syt10	−0.579	0.056
	Lrrtm1	−0.314	0.077
	Egr4	−0.542	0.080
	Sema5b	0.295	0.117
	Slitrk6	−0.831	0.117
	Htrlb	−0.530	0.120
	Rgs2	−0.403	0.155
	Kcnj2	−0.392	0.163
	Gabrq	−1.310	0.163
	Nmbr	2.365	0.163
	Kcnj5	−0.742	0.166
	Sytl4	−1.556	0.169
	Calb1	−0.541	0.176
	Serinc2	1.516	0.193
	Pde11a	−0.472	0.193
M_IVSA_NAc	Nptxr	0.479	6.15E-05***
	Nptx2	0.417	0.003**
	Lingo1	0.302	0.063
	Ucn	1.923	0.072
M_IVSA_VTA	Cacna2d1	−0.268	0.009**
	Pde1c	0.356	0.039*
	Egr1	−0.426	0.058
F_IVSA_VTA	Calb1	0.334	0.091

Log_2_FC and FDR values for candidate genes of interest described in Section 3.2. All listed genes met thresholds of |log_2_FC| > 0.25 and FDR < 0.2. *FDR < 0.05 **FDR ≤ 0.01 ***FDR ≤ 0.001.

### 3.3 Adolescent OXY induced differential alternative splicing in the adult NAc and VTA

It has been established that cocaine intake and abstinence induce differential alternative splicing, which often outnumbers differential gene expression (Feng et al., 2014). We have previously leveraged Multivariate Analysis of Transcript Splicing (rMATS) (Shen et al., 2014) to identify cocaine-induced alternative splicing events that, when manipulated with gene-targeted epigenetic editing, were sufficient to modulate cocaine self-administration (Xu et al., 2021). Global alternative splicing analysis has rarely been reported in opioid-related studies. Here, we applied rMATS to identify differential splicing in mice that received adolescent OXY vs. SAL with or without IVSA. RNA-seq was performed at a depth of approximately 50 million reads per sample to facilitate this analysis (library depth statistics provided in Supplementary Table 1).

The total number of detected events (≥10 junction counts in at least *n* = 3 libraries within each dataset) vs. statistically significant events, as well as FDR and dPSI distribution statistics, are provided in Supplementary Table 4. Skipped Exon (SE) events were more prevalent than all other possible event types (Retained Intron [RI], Mutually Exclusive Exon [MXE], Alternative 5’ Splice Site [A5SS], and Alternative 3’ Splice Site [A3SS]), which is expected based on prior work (Murphy et al., 2026; Krick et al., 2025; Xu et al., 2021; Hu et al., 2020; Hu et al., 2017). Volcano plots of SE events are shown in Figure 3A (NAc) and Figure 3B (VTA), with other events shown in Supplementary Figure 3A. rMATS output statistics for the complete lists of significant events for each dataset are provided in Supplementary Table 4. While having a comparable number of total detectable events to all other datasets, F_IVSA_VTA exhibited the greatest number of DSGs with 387 across all event types, more than twice as many as any other dataset. There was no overlap between differentially spliced genes (DSGs) identified by rMATS and candidate genes identified by DESeq2 in any dataset (Figures 3C,D), consistent with our previous findings that drugs of abuse and other stimuli regulate expression and alternative splicing in distinct gene sets (Xu et al., 2021; Hu et al., 2017; Krick et al., 2025). Most DSGs were unique to individual datasets (Figures 3E,F), with relatively few shared across datasets. Our initial hypothesis, which was not supported by our DESeq2 analysis, was that the female NAc or VTA would exhibit a distinct differential gene expression signature after adolescent OXY that could potentially explain the observed increase in IVSA. In conjunction with our IVSA data, this alternative splicing analysis leads us to a new hypothesis that differential splicing in the female VTA during opioid re-exposure following adolescent OXY could drive the increased remifentanil IVSA that we observed.

**Figure 3:**
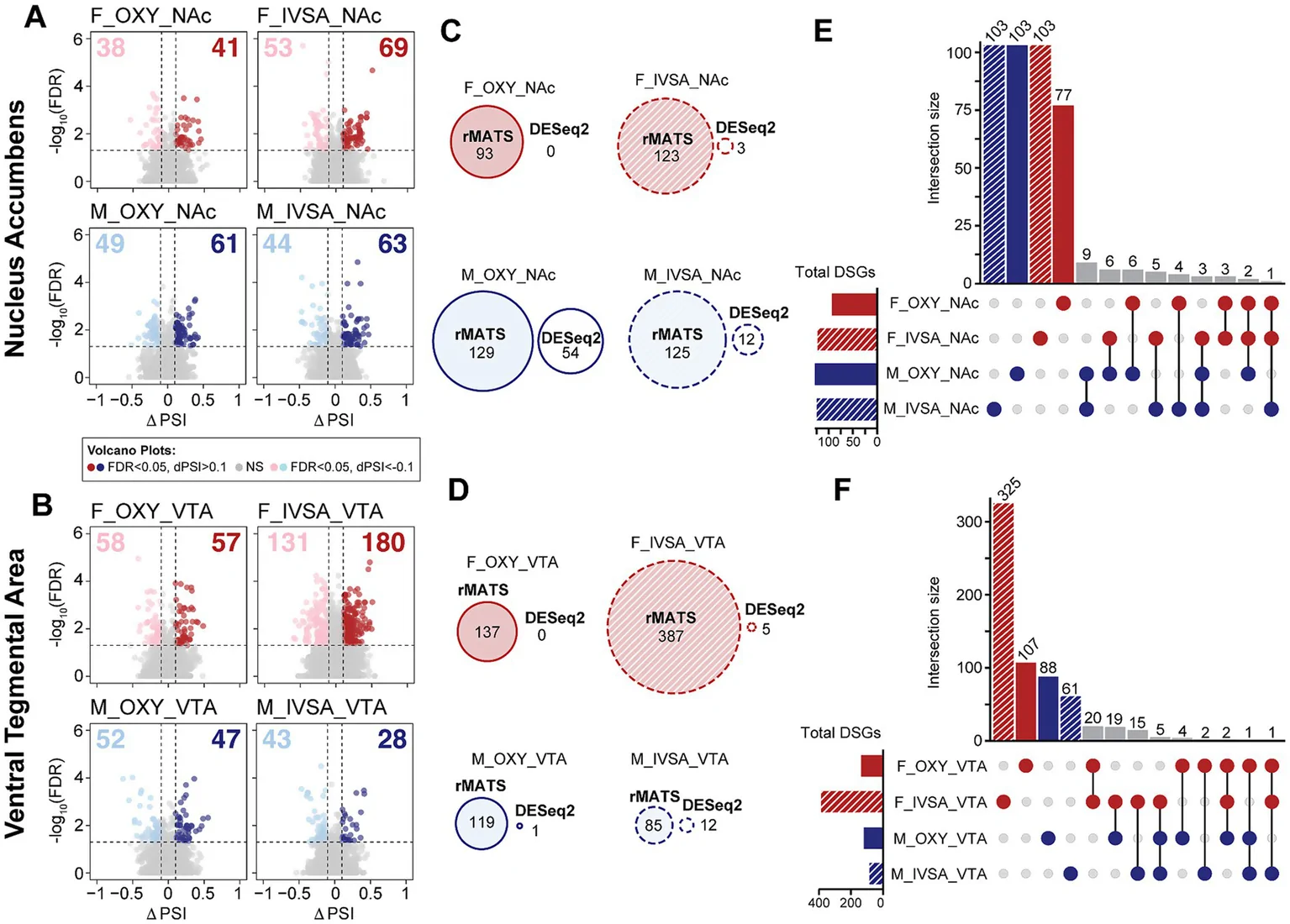
Adolescent OXY induced differential splicing, especially in the VTA of female mice that underwent IVSA. **(A,B)** Volcano plots of dPSI vs. -log_10_FDR for Skipped Exon events computed by rMATS in the NAc **(A)** and VTA **(B)**. Highlighted events meet cutoffs of FDR < 0.05 and dPSI >|0.1|. **(C,D)** Euler plots of genes identified by rMATS (FDR < 0.05, dPSI >|0.1|) vs. DESeq2 (FDR < 0.2, |log_2_FC| > 0.25) analysis in the NAc **(C)** and VTA **(D)**. DSG lists include genes with at least one significant event across all possible event types. **(E,F)** Upset plots of DSGs across datasets in the NAc **(E)** and VTA **(F)**. Total DSGs are indicated by the horizontal bars on the left, and intersections of DSGs by the vertical bars. Solid blue = M_OXY, solid red = F_OXY, striped blue = M_IVSA, striped red = F_IVSA, gray = overlap of any two or more datasets.

The DSGs identified here require further validation, for example, by RT-PCR with primers flanking the putative alternative splicing events (Krick et al., 2025), but the data presented here provide a number of compelling targets for our future work. For example, F_IVSA_VTA exhibited increased usage of an alternative 5′ splice site within exon 2 of *brain-derived neurotrophic factor* (*Bdnf*), an IEG with well-characterized roles in the neural response to drugs of abuse. Specifically, there was increased usage of an alternative 5′ splice site within Exon 2 (*Bdnf* 2C vs. 2B; dPSI = 0.214, FDR = 0.010; Supplementary Figure 3B), which has been found to influence dendritic localization of the mRNA (Colliva and Tongiorgi, 2021). F_OXY_NAc and M_OXY_VTA both exhibited differential splicing of *cAMP response element modulator* (*Crem*), a component of the transcriptional response downstream of cyclic AMP signaling, which is regulated by opioid exposure and withdrawal (Terwilliger et al., 1991; Ammon-Treiber et al., 2004; Wang et al., 2007) and has two distinct protein isoforms, CREM and ICER (Mioduszewska et al., 2003). Specifically, F_OXY_NAc had increased inclusion of a CREM-specific exon (dPSI = 0.225, FDR = 0.015, Supplementary Figure 3C), while M_OXY_VTA had decreased inclusion of an exon present in both proteins (dPSI = −0.156, FDR = 0.034, Supplementary Figure 3D). F_OXY_VTA and M_OXY_NAc both exhibited differential splicing of *Neurexin-1* (*Nrxn1*), a critical protein for synaptic structure that has been implicated in disorders including autism and schizophrenia. Specifically, F_OXY_VTA had increased inclusion of *Nrxn1* exon 4 (dPSI = 0.268, FDR = 0.050, Supplementary Figure 3E), while M_OXY_NAc exhibited decreased alternative 5′ splice site usage at exon 7 (dPSI = −0.121, FDR = 0.005, Supplementary Figure 3F), both components of *Nrxn1α* isoforms (Cooper et al., 2024). Junction counts for these events are shown in Supplementary Figure 3.

### 3.4 Gene-set enrichment analysis revealed term enrichment associated with adolescent OXY in the adult NAc and VTA

Next-generation sequencing has enabled the rapid generation of deep RNA-seq datasets that facilitate genome-wide target discovery. That said, interpreting changes in biological function from these datasets is challenging due to noise introduced by inter-animal biological variability and batch effects from tissue processing and RNA sequencing. Furthermore, single-gene analysis may miss effects on broader biological pathways, as single genes do not act in isolation to influence cell function (Subramanian et al., 2005). One common approach to extract biological function from sequencing data is to perform Overrepresentation Analysis (ORA) on DEG lists compared to established sets of genes with related biological functions, such as Gene Ontology terms (Ashburner et al., 2000). This approach fails when many individual DEGs are not identified, as in the current study, which can occur when the signal is outweighed by noise or when functional changes are driven by small expression changes in many related genes. Therefore, we applied threshold-free gene-set enrichment analysis (Subramanian et al., 2005; Xu et al., 2024; Wu et al., 2021) to identify enrichment of Gene Ontology (GO) terms in OXY vs. SAL groups using complete gene expression data without applying log_2_FC and FDR cutoffs.

GSEA was performed using all three GO aspects (Molecular Function [MF], Biological Process [BP], and Cellular Component [CC]), with genes ranked by the signed Wald statistic generated by DESeq2. Despite the limited individual differential gene expression we observed, all datasets exhibited some significant term enrichment (FDR < 0.05), but notable differences emerged in the number of enriched terms across datasets. GSEA output statistics for all significantly enriched terms are provided in Supplementary Table 5. F_OXY_NAc, which had 0 genes meeting our DESeq2 threshold of FDR < 0.2, also had minimal significant term enrichment, with 0 MF, 1 BP, and 9 CC terms meeting our GSEA threshold of FDR < 0.05 (Figures 4A–C). In contrast, F_IVSA_NAc, which had just 3 candidate genes with FDR < 0.2, exhibited substantial term enrichment, with 52 MF, 104 BP, and 36 CC terms meeting the GSEA FDR cutoff. M_OXY_NAc, which exhibited the greatest number of individual DEGs, also had the greatest number of enriched GO terms in the NAc (52 MF, 221 BP, 87 CC), followed by M_IVSA_NAc (39 MF, 206 BP, 57 CC). Though differential gene expression was minimal in all VTA datasets, GSEA revealed substantial significant term enrichment at the OXY timepoint in both males and females (F_OXY_VTA: 119 MF, 455 BP, 160 CC; M_OXY_VTA: 70 MF, 349 BP, 125 CC), but very limited term enrichment at the IVSA timepoint (F_IVSA_VTA: 2 MF, 12 BP, 24 CC; M_IVSA_VTA: 6 MF, 3 BP, 6 CC).

**Figure 4:**
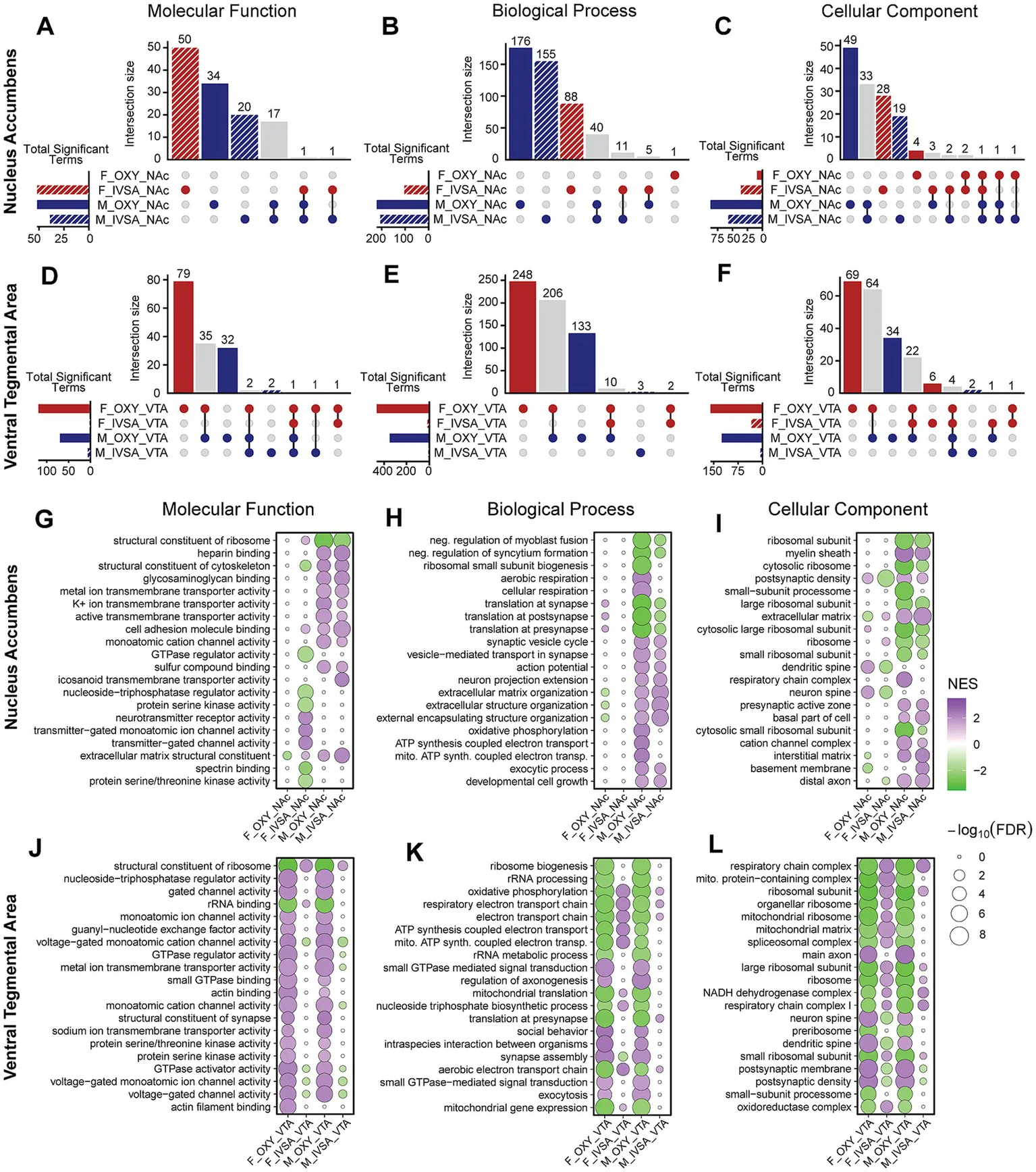
Gene-set enrichment analysis revealed term enrichment associated with adolescent OXY in the NAc and VTA. **(A–F)** Upset plots of significantly enriched GO terms calculated by GSEA (clusterProfiler::gseGO) within Molecular Function **(A,D)**, Biological Process **(B,E)**, and Cellular Component **(C,F)** ontologies for NAc **(A-C)** and VTA **(D–F)**. Total significant terms are indicated by horizontal bars, and intersections by vertical bars. Solid blue = M_OXY, solid red = F_OXY, striped blue = M_IVSA, striped red = F_IVSA, gray = overlap of any two or more datasets. **(G–L)** Dot plots of the top 20 significantly enriched GO terms across datasets within NAc **(G–I)** and VTA **(J–L)**, sorted by the number of significant datasets * absolute Normalized Enrichment Score (NES) * -log_10_FDR. Dot size corresponds to –log_10_FDR, and color corresponds to NES computed by GSEA.

Dot plots of the top five most significant positively and negatively enriched terms for each dataset, ranked by FDR, are shown in Supplementary Figure 5. While many enriched terms were unique to a single dataset, a large proportion of those enriched in the NAc overlapped between M_OXY_NAc and M_IVSA_NAc, with little overlap between males and females (Figures 4A–C). In contrast, in the VTA, the greatest overlap was between F_OXY_VTA and M_OXY_VTA, with very little overlap between OXY and IVSA timepoints (Figures 4D–F). To visualize patterns of functional enrichment between datasets, GSEA results were compiled by brain region, filtered for pathways with FDR < 0.05 in at least one dataset, and sorted based on the number of significant datasets * absolute Normalized Enrichment Score (NES) * -log_10_FDR. The top 20 terms using this sorting strategy for MF, BP, and CC are shown in Figures 4G–L. The magnitude and direction of the Normalized Enrichment Score (NES) for these terms were highly correlated between M_OXY_NAc and M_IVSA_NAc (Figures 4G–I). This included terms related to ion transmembrane transporter activity, synaptic translation, and extracellular organization. In contrast, F_IVSA_NAc showed enrichment of terms related to neurotransmitter-gated channel activity. Among the few GO terms shared between males and females, including Structural Constituent of Ribosome and Structural Constituent of Cytoskeleton, enrichment occurred in opposite directions, as indicated by opposing NES values. F_OXY_VTA and M_OXY_VTA (Figures 4J–L) had overlapping enrichment of terms related to voltage-gated ion channel activity, as well as mitochondrial activity and energy synthesis. As in the NAc, several terms related to synaptic translation were also enriched. Although enrichment was limited at the IVSA timepoint, significant terms tended to overlap with those at the OXY timepoint, including those related to voltage-gated channel activity, the electron transport chain, and synaptic translation. For all those shared terms, the direction of NES was consistent between males and females but reversed between the OXY and IVSA timepoints.

As with our DESeq2 analyses, conclusions drawn from these data are limited by the confound of sex, brain region, and timepoint with batch. Visual patterns of enrichment between datasets cannot be tested statistically but can be used to generate new hypotheses for future testing. Taken together, these GSEA results lead us to a new hypothesis that while the female NAc shows minimal individual gene expression differences between OXY and SAL groups at the OXY timepoint, prior adolescent OXY exposure may alter the functional enrichment of RNA expression in the female NAc after subsequent remifentanil IVSA. In contrast, we hypothesize that the male NAc exhibits transcriptional enrichment to adolescent OXY that is distinct from females and is similar with or without the subsequent experience of IVSA. In the VTA, both male and female mice may exhibit functional transcriptomic enrichment associated with adolescent OXY alone, which is no longer present after remifentanil IVSA.

Given the limited number of individual DEGs detected by DESeq2, it is important to consider the underlying patterns of gene expression contributing to GO term enrichment in GSEA. To this end, CNET plots were generated to visualize the log_2_FC of constituent genes for the GO::MF terms shown in Figure 4 (Supplementary Figures 5, 6). GSEA enrichment plots for key terms in the NAc and VTA are shown in Supplementary Figures 7, 8. These plots illustrate that significant enrichment was driven by small but directionally consistent changes across numerous genes associated with these GO terms. For example, in F_IVSA_NAc, Neurotransmitter Receptor Activity was significantly enriched through small increases in several GABA receptor subunit (*Gabr*), glycine receptor subunit (*Glra*), and glutamate receptor subunit (*Gri*) genes. In contrast, M_OXY_NAc and M_IVSA_NAc exhibited small increases in numerous potassium channel subunit (*Kcn*), calcium channel subunit (*Cacn*), and solute carrier (*Slc*) genes, contributing to ion channel and transmembrane transporter pathway enrichment.

For increased rigor, we considered the possibility that neuronal function-related terms are significantly enriched by random chance, given that the sequenced RNA is brain-specific. This concern was addressed in part by permutation tests inherent to the gseGO algorithm. We further addressed this by running a randomized control GSEA, in which the order of gene symbols to Wald statistic values was randomized. In every dataset, this randomized control test yielded zero significant pathways by FDR, lending validity to our findings.

Broader functional analysis of differential splicing is more challenging than differential expression because the more or less inclusion of a particular splicing event is not as meaningful as the function of the spliced sequence. GO ORA can be performed on DSG lists to elucidate term enrichment (Murphy et al., 2026; Krick et al., 2025). However, in the current analysis, little to no term enrichment was detected in any dataset, even in F_IVSA_VTA, despite its relatively high number of DSGs. We next performed threshold-free GSEA on all rMATS events passing our junction read threshold. This approach is less common with rMATS output, as ranking of DSGs is not intuitive since dPSI is bounded to (−1,1). In this case, we chose to rank events by absolute dPSI * -log_10_(FDR). Similar to the ORA, this approach did not reveal any functional enrichment.

### 3.5 Rank-rank hypergeometric overlap revealed concordance in the transcriptional response to adolescent OXY in the NAc and VTA

As a complement to GSEA, we applied stratified rank-rank hypergeometric overlap (RRHO2) (Cahill et al., 2018) to assess patterns of global gene expression across datasets. This is done by performing a stepwise comparison of sets of ranked genes in each dataset and computing whether these gene lists overlap more than expected by chance. As with GSEA, gene expression data were analyzed without filtering by log_2_FC or FDR, with genes ranked by the Wald statistic computed by DESeq2. In the resulting heatmaps, higher -log_10_(p) values in the top right and bottom left quadrants reflect dataset concordance (i.e., genes that are elevated in one dataset are also elevated in the other), while higher -log_10_(p) values in the bottom right and upper left quadrants reflect dataset discordance (genes that are elevated in one dataset are decreased in the other; Figure 5A). To facilitate comparison of the degree of concordance across datasets, heatmaps were generated with the color scale for -log(p) normalized within brain region. The maximum color value was limited to the 90th percentile of the overall maximum to preserve the dynamic range of the scale at lower values. Raw RRHO2 heatmap output is shown in Supplementary Figure 9A.

**Figure 5:**
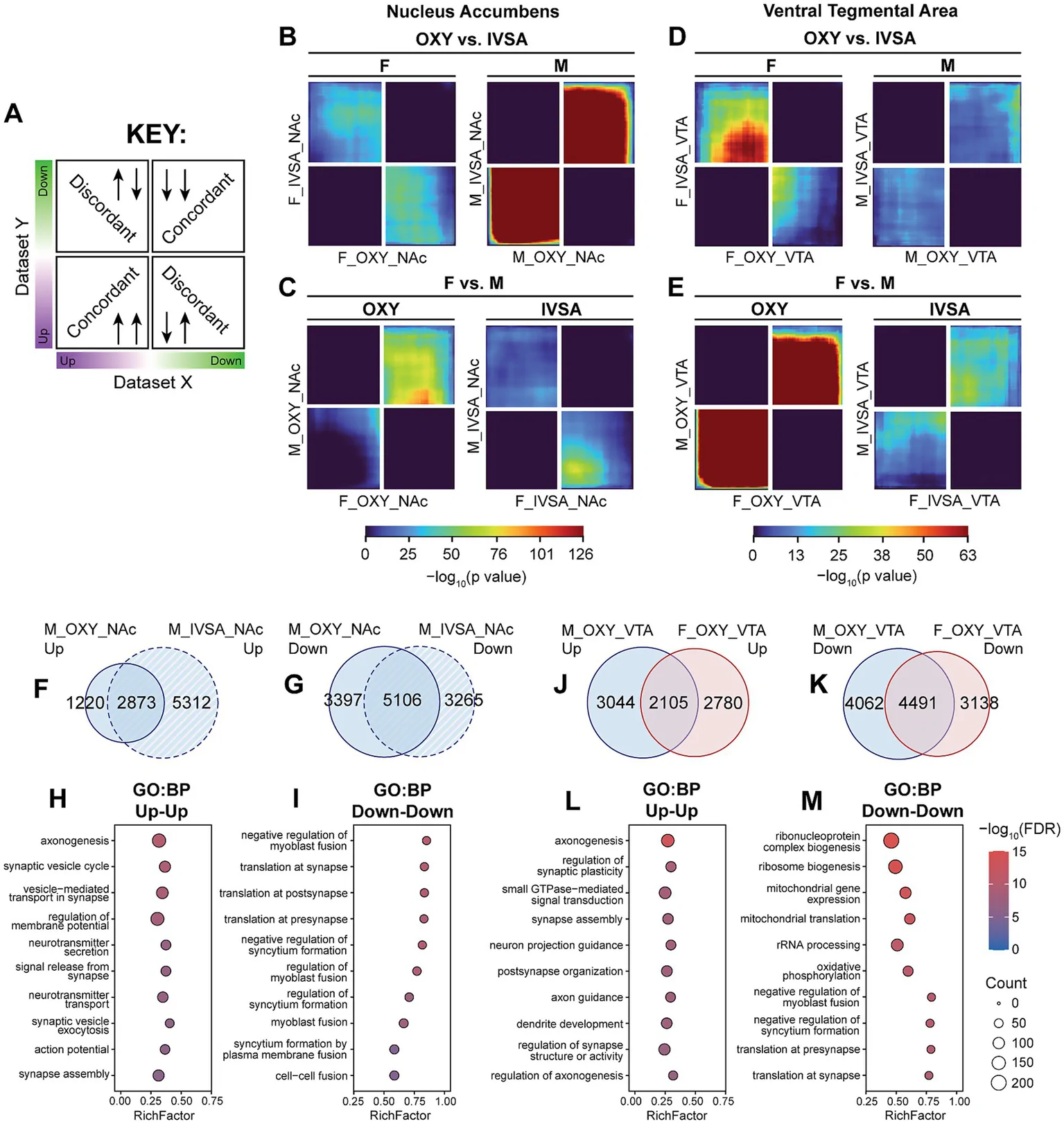
Rank-rank hypergeometric overlap revealed concordance in the transcriptional response to adolescent OXY in the NAc and VTA. **(A)** Key for interpretation of RRHO heatmaps. Gene expression data were ranked by the Wald statistic computed by DESeq2. More extreme values on the *x*- and *y*-axes correspond to larger gene expression changes in OXY vs. SAL. Higher -log_10_(p) values, indicated by the heatmap color scale, correspond to greater concordance (upper right and lower left quadrants) or discordance (upper left and lower right quadrants) in gene expression changes between the X and Y datasets. **(B)** RRHO heatmaps for comparisons between OXY and IVSA datasets within each sex in the NAc. F_OXY_NAc was mildly discordant with F_IVSA_NAc. M_OXY_NAc was highly concordant with M_IVSA_NAc. **(C)** RRHO heatmaps for comparisons between males and females within each timepoint in the NAc. F_OXY_NAc was mildly concordant with M_OXY_NAc. F_IVSA_NAc was mildly discordant with M_IVSA_NAc. **(D)** RRHO heatmaps for comparisons between OXY and IVSA datasets within each sex in the VTA. F_OXY_VTA was moderately concordant with F_IVSA_VTA. M_OXY_VTA was mildly concordant with M_IVSA_VTA. **(E)** RRHO heatmaps for comparisons between males and females within each timepoint in the VTA. F_OXY_VTA was strongly concordant with M_OXY_VTA. F_IVSA_VTA was mildly discordant with M_IVSA_VTA. The RRHO heatmap color scale was normalized across comparisons within each brain region, with the maximum color value limited to 90% of the overall maximum. Heatmaps of raw RRHO output without normalization are shown in Supplementary Figure 8A. **(F–I)** Euler plots of genes contributing to the most significant overlap in the Up-Up **(F)** and Down-Down **(G)** quadrants of M_OXY_NAc vs. M_IVSA_NAc, and the top 10 significantly enriched GO:: BP terms by ORA for the overlapping Up-Up **(H)** and Down-Down **(I)** genes. **(J–M)** Euler plots of genes contributing to the most significant overlap in the Up-Up **(J)** and Down-Down **(K)** quadrants of F_OXY_VTA vs. M_OXY_VTA, and the top 10 significantly enriched GO::BP terms by ORA for the overlapping Up-Up **(L)** and Down-Down **(M)** genes. Gene lists for Euler plots are provided in Supplementary Table 6. Full GO ORA results are provided in Supplementary Table 7.

There was strong concordance in the NAc between M_OXY_NAc and M_IVSA_NAc (Figure 5B, right), consistent with our observation that these datasets have overlapping and positively correlated gene-set enrichment (Figures 4A–C,G). F_OXY_NAc and M_OXY_NAc showed weaker concordance (Figure 5C, left). F_IVSA_NAc was discordant with both F_OXY_NAc (Figure 5B, left) and M_IVSA_NAc (Figure 5C, right), consistent with our observation that the male and female NAc exhibit opposing gene-set enrichment in response to remifentanil IVSA following adolescent OXY. In the VTA, RRHO revealed strong concordance between gene expression in F_OXY_VTA and M_OXY_VTA (Figure 5E, left), consistent with the substantial GSEA overlap between these datasets (Figures 4D–F,J). F_IVSA_VTA and M_IVSA_VTA showed weaker positive concordance, also consistent with their more limited GSEA overlap (Figure 5E, right). F_OXY_VTA and F_IVSA_VTA showed moderate discordance (Figure 5D, left), while M_OXY_VTA and M_IVSA_VTA showed mild concordance (Figure 5D, right).

For increased rigor, we further used RRHO2 to compute the odds ratio of ranked gene overlap, which is less likely than the hypergeometric test to be skewed by comparing large numbers of genes (Cahill et al., 2018). Odds ratio heatmaps for all datasets are presented in Supplementary Figure 9B. Interpretation of the resulting heatmaps is the same with regard to concordance and discordance. In all cases, the pattern of concordance agreed with that of the hypergeometric heatmaps, supporting that our findings are not skewed by the inclusion of all overlapping expressed genes (~17–18,000 genes per dataset). Additionally, as with the GSEA, we performed a randomized control analysis with gene symbols scrambled relative to the ranking statistic. Randomized heatmaps are shown in Supplementary Figure 9C. In all cases, randomization abolished the concordance patterns in the original analysis, supporting that these results are not likely false positives due to all RNA being from brain tissue.

For the two comparisons with the strongest concordance, M_OXY_NAc vs. M_IVSA_NAc and F_OXY_VTA vs. M_OXY_VTA, the genes contributing to the most significant hotspots in the up-up and down-down quadrants were extracted from the RRHO2 results (Emerson et al., 2023). Gene lists are provided in Supplementary Table 6. In the male NAc, the most significant hypergeometric overlap occurred in the up-up quadrant when comparing the top 4,093 genes in M_OXY_NAc and the top 8,185 genes in M_IVSA_NAc. A total of 2,873 genes overlapped in this comparison (Figure 5F). In the down-down quadrant, the most significant hypergeometric overlap occurred when comparing the bottom 8,503 genes in M_OXY_NAc and the bottom 8,371 genes in M_IVSA_NAc, resulting in 5,106 overlapping genes (Figure 5G). GO ORA analysis was performed on these overlapping gene lists. Importantly, terms significantly enriched in the up-up gene list (Figure 5H) overlapped with terms positively enriched by GSEA in both M_OXY_NAc and M_IVSA_NAc, including Vesicle-Mediated Transport in Synapse and Action Potential (Figure 4H). Terms significantly enriched in the down-down GO analysis (Figure 5I) overlapped with negatively enriched overlapping GSEA terms, including Translation at Synapse (Figure 4H). ORA output statistics for all significant RRHO GO terms are provided in Supplementary Table 7.

In the VTA at the OXY timepoint, the most significant hypergeometric overlap in the up-up quadrant was observed when comparing the top 5,149 genes in M_OXY_VTA and the top 4,885 genes in F_OXY_VTA, with 2,105 overlapping genes (Figure 5J). In the down-down quadrant, the most significant hypergeometric overlap was found when comparing the bottom 8,553 genes in males and the bottom 7,629 genes in females, resulting in 4,491 overlapping genes (Figure 5K). As in the NAc, enriched GO terms in these overlapping gene lists recapitulated the GSEA overlap between these datasets. Specifically, the up-up overlap was enriched for terms including Axonogenesis, Synapse Assembly, and Small GTP-ase Signal Transduction (Figure 5L), and the down-down overlap included terms such as Ribosome Biogenesis, Translation at Presynapse, and Mitochondrial Gene Expression (Figure 5M).

## 4 Discussion

Here, we present a novel finding that female mice exhibit increased remifentanil self-administration on a fixed-ratio schedule following adolescent OXY compared to SAL controls, while male mice do not. We also present RNA-seq datasets from two key reward-related brain regions, the NAc and VTA, at two timepoints: one coinciding with the first session of remifentanil IVSA (OXY vs. SAL; ‘OXY’), and the other immediately following the final session of IVSA (OXY + REMI vs. SAL+REMI, ‘IVSA’).

While adolescent OXY increased remifentanil intake (fixed-ratio response) in female mice, it did not impact progressive ratio responding or breakpoint in males or females. As these two schedules measure different behaviors, they can dissociate for several reasons. Low-ratio FR responding (FR1, FR2) costs almost no effort and reflects how much drug the animal consumes to maintain brain levels in a preferred range. This could be impacted, for example, by lasting tolerance, causing each infusion to produce less effect and requiring more infusions to achieve the same subjective state. PR, on the other hand, tracks how much work the animal will do to obtain one infusion, which can reflect motivation and the drug’s reinforcing efficacy (Madsen and Brown, 2022). The data presented here thus demonstrate a change in the regulation of intake, but not a change in motivation or reinforcing efficacy.

In contrast to our behavioral results, we observed few differentially expressed genes in the NAc or VTA at either timepoint but did identify several candidate genes of interest for future study (FDR < 0.2), particularly in the male NAc, including some that met the more stringent cutoff of FDR < 0.05 (Table 2). Together with our behavioral results, we hypothesize that the response of these genes to adolescent OXY in the male NAc could help promote control-like remifentanil self-administration. In addition to our differential expression analysis, alternative splicing analysis revealed numerous differential splicing events in all comparisons, especially in the female VTA post-IVSA, which we hypothesize could promote increased remifentanil self-administration after adolescent OXY.

Despite the limited differential gene expression identified by DESeq2 analysis, threshold-free Gene-set enrichment analysis (GSEA) revealed highly correlated enrichment of GO terms in the male NAc between the OXY and IVSA timepoints, driven by small but directionally consistent changes in many genes related to terms highly relevant to neuronal function. These include transmembrane transport, cation channel activity, and synaptic translation-related terms. Females exhibited minimal gene-set enrichment at the OXY timepoint but robust term enrichment at the IVSA timepoint. Notably, while this also occurred in neuron-related functional terms, these either did not overlap with those enriched in the male NAc or had opposing Normalized Enrichment Scores. Females specifically showed positive enrichment of terms such as Neurotransmitter Receptor Activity and Transmitter-Gated Channel Activity. Complementary RRHO analysis further revealed concordance between these datasets consistent with their overlapping gene set enrichment. GO Overrepresentation Analysis on the genes contributing to the most significant RRHO overlaps recapitulated many of the enriched terms from GSEA.

Many of the candidate genes of interest identified by this analysis are strongly related to neuronal function and make compelling targets for validation and manipulation in future work to test our new hypotheses. A particularly compelling target is Slc6a13, encoding GABA transporter 2 (GAT2), which was upregulated in the male NAc at the OXY timepoint (FDR = 3.61E^−5^, log_2_FC = 1.18). The GABA transporter family moves the inhibitory neurotransmitter GABA and its analog taurine across cell membranes. While GAT1 and GAT3 are expressed more highly in neurons, GAT2 may be important in moving GABA and taurine over the blood–brain barrier to modulate the GABA system (Takanaga et al., 2001; Zhou et al., 2012; Clausen et al., 2006). Notably, the taurine system is already targeted by the drug Acamprosate (N-Acetyl homotaurine) for the treatment of relapse in alcohol-use disorder (Kunwar and Risal, 2025), but it does not seem to have been targeted in opioid-use disorder. Other compelling targets, identified by both DESeq2 and GSEA analyses include potassium channel genes (such as *Kcnj5* and *Kcnj2*, both identified in M_OXY_NAc) and calcium-binding genes (such as *Calb1*, decreased in M_OXY_NAc and increased in F_IVSA_VTA).

We have previously shown that this adolescent OXY paradigm increases morphine conditioned place preference (CPP) in adolescent but not adult male mice (Sanchez et al., 2016), which was not tested in females. Other researchers have also demonstrated long-term effects of adolescent opioid exposure on CPP (Zhang et al., 2016; Madison et al., 2020). While the current results may seem to conflict with this literature, IVSA and CPP are behavioral paradigms that test distinct aspects of drug-related behavior—reinforcement vs. reward—which may diverge (Green and Bardo, 2020). Furthermore, while IVSA relies on operant learning, CPP relies on spatial learning, which are regulated by different brain regions for example, the frontal cortex for operant (Cetin et al., 2004) and the hippocampus for spatial (Bird and Burgess, 2008). These regions, which were not profiled here, could also have sex and age-specific responses to adolescent oxycodone exposure and abstinence.

The VTA showed almost no differential gene expression across all comparisons. However, both the male and female VTA at the OXY timepoint showed robust gene-set enrichment of brain function-related GO terms that were consistent in direction and magnitude of enrichment between the two sexes. Specifically, we observed significant enrichment of terms related to voltage-gated ion channel activity and ion transmembrane transport, suggesting axonal processes and changes in presynaptic signal transmission. In the IVSA cohort, gene-set enrichment in the VTA was minimal. In some cases, the direction of enrichment was opposite in the OXY and IVSA cohorts. For example, Structural Constituent of Ribosome was negatively enriched in both males and females at the OXY timepoint but positively enriched in both sexes post-IVSA. Together, these patterns suggest that the VTA potentially has a functionally significant gene expression response to adolescent OXY that is similar in both males and females.

In another of our prior studies, also using this OXY paradigm, we quantified several dopamine signaling-related genes (*Cartpt, Dat, Th, Nr4a2*) in the VTA and found that they were elevated after adult OXY exposure but failed to activate after adolescent OXY in male mice (Carpenter et al., 2021). Consistent with these findings, our current results show that neither male nor female mice have substantial individual differential gene expression in the VTA after adolescent OXY, including in the four genes previously examined by qPCR. This suggests that both male and female adolescent mice may have a reduced homeostatic transcriptional response to OXY in the VTA compared to adults, though this comparison is limited by the lack of an RNA-seq dataset for adults with this paradigm. In contrast, males do appear to mount a homeostatic transcriptional response in the NAc, though how this compares to that of the adult NAc in this paradigm is unknown.

In addition to profiling differential gene expression, we also analyzed differential alternative splicing using rMATS (Shen et al., 2014). As we and others have previously reported with cocaine (Xu et al., 2021; Feng et al., 2014), we observed robust differential alternative splicing in all datasets, which did not overlap with candidate genes identified by differential expression analysis, suggesting that these changes could reflect parallel but distinct regulatory pathways. The largest number of DSGs by far was identified in the female VTA post-IVSA. While the confound of sex, brain region, and timepoint with batch limits our conclusions, we hypothesize that this could represent a maladaptive response that drives increased remifentanil IVSA after adolescent OXY. Minimal functional enrichment was detected by overrepresentation analysis of GO terms in any dataset. While inconclusive, this result is not altogether surprising; this approach is limited in its ability to characterize the functional consequences of alternative splicing, as greater or lesser inclusion of a particular splicing event does not necessarily correspond to the direction of its functional effect. Rather, the consequence depends on the specific functional or regulatory domains affected by the inclusion or exclusion of the alternatively spliced sequence. That said, several DSGs were genes with functional splice variants that are known to be regulated by opioid exposure. For example, F_IVSA_VTA exhibited differential splicing of *brain-derived neurotrophic factor* (*Bdnf*), an IEG with well-characterized roles in synaptic plasticity and the neural response to drugs of abuse. Specifically, there was increased usage of an alternative 3′ splice site within Exon 2 (*Bdnf* 2C vs. 2B), which may influence dendritic localization of the mRNA (Colliva and Tongiorgi, 2021). *Bdnf* exon 2 variants have previously been reported as upregulated by morphine CPP (Meng et al., 2013), though differential usage of 2C vs. 2B was not reported. F_OXY_NAc and M_OXY_VTA both exhibited differential splicing of *cAMP response element modulator* (*Crem*), a component of the transcriptional response downstream of cyclic AMP signaling, which is regulated by opioid exposure and withdrawal (Terwilliger et al., 1991; Ammon-Treiber et al., 2004; Wang et al., 2007). This gene produces two functional proteins: CREM, a transcriptional activator, and ICER, a repressor (Mioduszewska et al., 2003). Specifically, F_OXY_NAc had higher inclusion of the 3rd exon, a component specifically of CREM transcripts, and M_OXY_VTA of the 10th, associated with both CREM and ICER transcripts (Sánchez-Jasso et al., 2023), though the specific function of these two exons is unclear. All of these events make compelling targets for validation, for example, by RT-PCR, and for follow-up study.

A strength of the current study is the inclusion of multiple sexes, brain regions, and timepoints. That said, a considerable limitation of bulk RNA-seq is that the statistical power to detect DEGs is reduced by sources of variability, such as inter-animal biological variation, cellular heterogeneity within bulk tissue, and batch effects introduced during sample processing. Careful experimental design and consistency may minimize, but not eliminate, these effects, particularly in the current study, in which bulk brain tissue was collected from multiple groups of mice over a protracted experimental timeline. Sample processing was designed to maximize our power to compare OXY vs. SAL treatment. We leveraged several existing bioinformatics tools to compare patterns of change across sexes, brain regions, and timepoints to identify meaningful patterns. However, we could not directly statistically compare factors besides treatment with DESeq2 since they are confounded with sequencing batches. While the analyses presented here have generated several new hypotheses for future study, our conclusions from these data are limited and require additional testing in future work to validate.

An additional consideration is that both the NAc and VTA are regions with diverse cell types. Namely, the VTA contains distinct populations of GABAergic cells, which are directly inhibited by opioids, and glutamatergic cells, which are regulated by GABA inhibition to modulate downstream dopamine release (Conrad et al., 2024). The NAc contains several distinct populations of dopamine receptor-expressing cells, including D1 and D2 medium spiny neurons, which receive dopaminergic input from the VTA to gate motivated behaviors through the direct vs. indirect pathways (Chen et al., 2021). Recent single-cell opioid studies in adult mice (Browne et al., 2025) and rats (Reiner et al., 2022) have found robust cell type-specific responses in the NAc. Our GSEA analyses, which identified significant functional enrichment in our expression data driven by small changes across many related genes, suggest that we could be missing meaningful gene expression changes that are small in magnitude or highly variable in bulk tissue but could exhibit stronger, more consistent responses within individual cell populations. Finally, as we did not analyze more acute withdrawal timepoints, such as 1 day or 1 week, or more protracted timepoints, such as 3 or 6 months, we do not know whether males and females differ in their overall transcriptomic response to adolescent OXY or in the time course of this response.

Altogether, the data presented here increase our understanding of the long-term behavioral effects of adolescent oxycodone exposure in male and female mice. We have identified both individual genes and biological pathways that could potentially respond in the brain in a sex- and region-specific manner, generating new hypotheses and providing targets for validation and causal investigation in future studies.

## Data Availability

The original RNA-seq datasets presented in the study are publicly available on Gene Expression Omnibus (GSE351093).

## Supplementary material

SUPPLEMENTARY FIGURE 1 Flowcharts of experimental samples. **(A)** Flowchart of total mice enrolled in the study. *n* = 8 mice/sex/treatment were dissected for the “OXY” cohort, which underwent 28d minipump exposure followed by 28d drug-free before tissue dissection. An initial *n* = 12 mice/sex/treatment were allocated to the “IVSA” cohort, which underwent remifentanil IVSA following the drug-free period. After losses from jugular vein catheter (JVC) surgery complications or patency loss, final *n* = 9F OXY, 7F SAL, 11M OXY, and 10M SAL. **(B)** Flowchart of samples processed for RNA-seq. *n* = 4/treatment/batch was planned, with single mice processed for all NAc samples, and 2 mice pooled for all VTA samples. Three SAL samples were processed for F_IVSA_VTA due to 7 total mice finishing IVSA in this group. *N* = 3 samples were sequenced for M_OXY_VTA (OXY and SAL treatment) and M_IVSA_NAc (OXY treatment) due to technical issues during RNA extraction, resulting in poor RNA quality/yield. Post-sequencing, *n* = 1 OXY sample each was excluded from F_IVSA_NAc and F_IVSA_VTA due to aberrant gene expression patterns observed by PCA **(C,F)**, hierarchical clustering **(D,G)**, and sample-to-sample correlation **(E,H)** of the top 500 most variable genes, prior to subsequent differential expression and alternative splicing analysis. PCA for these datasets following sample exclusion is shown in Figure 2.

SUPPLEMENTARY FIGURE 2 Dispersion estimates and MA plots for all DESeq2 analyses. **(A–H)** Dispersion plots generated by deseq2::plotDispEsts. Dispersion estimates show the expected inverse relationship with the mean of normalized counts. Trends were relatively consistent across all datasets. **(I–P)** MA plots generated by deseq2::plotMA. Plots show a typical funnel shape centered around log fold change = 0, with higher variability in fold change estimates in genes with low expression.

SUPPLEMENTARY FIGURE 3 Differential splicing across all event types. **(A)** Volcano plots show dPSI vs. –log_10_FDR computed by rMATS for all event types. Highlighted significant events meet cutoffs of dPSI>|0.1| and FDR < 0.05. Skipped Exon (SE) events were the most common in all datasets, with fewer events detected for Retained Intron (RI), Mutually Exclusive Exon (MXE), Alternative 5’ Splice Site (A5SS), and Alternative 3’ Splice Site (A3SS). **(B–F)** Included Junction Counts (IJC) and Skipped Junction Counts (SJC) calculated by rMATS for select events of interest. **(B)** *Bdnf* A5SS, long exon: ch2:109506241–109506740, short exon: ch2:109506241–109506,657. **(C)** *Crem* Skipped Exon, ch18: 3325360–3325478. **(D)** *Crem* Skipped Exon, ch18: 3276693–3276729. **(E)** *Nrxn1* Skipped Exon, ch17: 91299542–91299572. **(F)** *Nrxn1* A5SS, long exon: ch17: 91009394–91009439, short exon: ch17: 91009415–91009439.

SUPPLEMENTARY FIGURE 4 Dot plots of top significantly enriched GO terms within each dataset. GO term enrichment was calculated by GSEA (clusterProfiler::gseGO). The top 5 positively enriched and top 5 negatively enriched terms, based on FDR ranking, are shown for each dataset, sorted by Normalized Enrichment Score (NES). Where fewer than 5 terms meet the cutoff of FDR < 0.05, all terms are shown, for example, F_OXY_NAc **(A)** has 0 MF, 1 BP, and 9 CC (4 positive, 5 negative) terms meeting the FDR cutoff. Dot size corresponds to the number of genes contributing to the enrichment (Gene set size), and color corresponds to -log_10_FDR, with scales normalized across all datasets within each brain region (NAc, **A–D**, and VTA, **E–H**).

SUPPLEMENTARY FIGURE 5 CNET plots of top GO::MF terms in the NAc. For Molecular Function GO terms shown in Figures 4G–I, CNET plots generated by clusterProfiler are presented. Only significant pathways (FDR < 0.05) are shown for each dataset. Circles for individual genes are colored by log_2_FC for OXY vs. SAL from DESeq2 results, with the color scale normalized across all plots.

SUPPLEMENTARY FIGURE 6 CNET plots of top GO::MF terms in the VTA. For Molecular Function GO terms shown in Figures 4J–L, CNET plots generated by clusterProfiler are presented. Only significant pathways (FDR < 0.05) are shown for each dataset. Circles for individual genes are colored by log_2_FC for OXY vs. SAL from DESeq2 results, with the color scale normalized across all plots.

SUPPLEMENTARY FIGURE 7 GSEA enrichment plots for select GO::MF terms in the NAc. All plots are labeled with Pathway, Dataset, NES, and FDR. Plots are shown for all NAc datasets for each pathway, regardless of whether FDR met the cutoff of <0.05, to demonstrate the difference between significant and non-significant enrichment patterns. **(A–D)** Structural constituent of ribosome. **(E–H)** Potassium ion transmembrane transporter activity. **(I–L)** Monoatomic cation channel activity. **(M–P)** Neurotransmitter receptor activity. **(Q–T)** Extracellular matrix structural constituent. **(U-X)** Transmitter−gated monoatomic ion channel activity.

SUPPLEMENTARY FIGURE 8 GSEA enrichment plots for select GO::MF terms in the VTA. All plots are labeled with Pathway, Dataset, NES, and FDR. Plots are shown for all VTA datasets for each pathway, regardless of whether FDR met the cutoff of <0.05, to demonstrate the difference between significant and non-significant enrichment patterns. **(A–D)** Structural constituent of ribosome. **(E–H)** Structural constituent of synapse. **(I–L)** Voltage-gated monoatomic ion channel activity. **(M–P)** Sodium ion transmembrane transporter activity.

SUPPLEMENTARY FIGURE 9 RRHO2 heatmaps for supplemental analyses. **(A)** Heatmaps output from RRHO2 hypergeometric tests with raw color scales. The maximum color scale within each heatmap reflects the maximum –log(p) for the hypergeometric test for that comparison. **(B)** Heatmaps of RRHO overlap computed by the Log Odds method. The color scale for log(Odds Ratio) was bounded to (−2,2). **(C)** Heatmaps of RRHO overlap from a control analysis in which gene symbols were randomized relative to the Wald statistic. To facilitate comparison with the original analysis, color scales were limited to the 90th percentile of the maximum –log(p) for the original NAc and VTA analyses, as shown in Figures 5B–E.

SUPPLEMENTARY TABLE 1 Library-level sequencing QC.

SUPPLEMENTARY TABLE 2 IVSA statistics.

SUPPLEMENTARY TABLE 3 DESeq2 statistics.

SUPPLEMENTARY TABLE 4 rMATS statistics.

SUPPLEMENTARY TABLE 5 GSEA statistics.

SUPPLEMENTARY TABLE 6 RRHO Euler plot gene lists.

SUPPLEMENTARY TABLE 7 RRHO GO ORA statistics.
